# Editorial: Dyadic Coping

**DOI:** 10.3389/fpsyg.2019.01498

**Published:** 2019-06-27

**Authors:** Guy Bodenmann, Mariana K. Falconier, Ashley K. Randall

**Affiliations:** ^1^Department of Psychology, University of Zurich, Zurich, Switzerland; ^2^School of Public Health, University of Maryland, Virginia Tech, Blacksburg, VA, United States; ^3^Counseling and Counseling Psychology, Arizona State University, Tempe, AZ, United States

**Keywords:** dyadic coping, couple support, spousal support, social support, emotion co-regulation

## Theoretical Background and Key Assumptions

Twenty years ago, dyadic coping arose from the conceptualization of stress and coping in romantic dyads, abandoning the traditional individual view of these phenomena. Since then theoretical contributions on dyadic coping (Bodenmann, 1997; Revenson et al., 2005; Revenson and Lepore, 2012; Falconier et al., 2016) and empirical studies have been published (see review article by Falconier and Kuhn), which extend work on conceptualizing dyadic coping and its beneficial association on individual and relational well-being.

Based on the individual-oriented theory of stress and coping by Lazarus and Folkman (1984), the Systemic Transactional Model (STM; Bodenmann, 1995, 2005) became an internationally recognized theory of stress and coping in couples and families. The key assumption of STM refers to the interdependence between romantic partners (Kelley et al., 1983), ascertaining that stress of one romantic partner always affects the other partner, through processes of stress spillover (Bodenmann et al., 2007) and cross-over (Neff and Karney, 2007). As such, this view of shared or mutual stress opens the possibility for common/joint dyadic coping, where either one partner supports the other in his/her own coping efforts (supportive or delegated dyadic coping) or both partners engage together in shared problem-solving or joint emotion-regulation (common dyadic coping).

Dyadic coping can be conceptualized as positive (emotion-focused, problem-focused, or delegated) or *negative* (e.g., superficial, ambivalent, hostile) (Bodenmann et al., 2016). From a STM perspective (Bodenmann, 1995, 2005), dyadic coping involves *cognitive* (individual and dyadic appraisals of stress and coping resources, individual and dyadic goals), *emotional* (shared emotions and co-regulation of emotions), *physiological* (shared arousals, impact of dyadic coping on endocrine processes) and *behavioral* aspects and processes (e.g., overt stress management activities, active listening to the partner's stress-related self-disclosure, verbal and non-verbal support behaviors like holding each other, hugging, giving a massage, active joint problem-solving). Dyadic coping is usually assessed by self-reports (such as e.g., the Dyadic Coping Inventory; DCI, Bodenmann, 2008), diary studies or behavioral coding (e.g., Kuhn et al., 2018; Leuchtmann et al., 2018; Lau et al.).

## Evolution of Dyadic Coping Research

Entering the keyword “dyadic coping” in ISI Web of Knowledge yields an impressive figure of the development of dyadic coping research since 1992. While in 1992, four publications cited dyadic coping, in 2018, 1,347 publications were referring to couple's coping.

At the beginning dyadic coping research focused primarily on the impact of *daily hassles* on couples' functioning (e.g., relationship satisfaction, couple's communication, sexuality, commitment, relationship dissolution). Researchers were interested in stress spill-over and cross-over processes within couples exposed to daily external stress and how couples effectively cope with these stressors (Story and Bradbury, 2004; Neff and Karney, 2007; Randall and Bodenmann, 2009, 2017; Falconier et al., 2015a,b). In the last decade, the field moved further into couples dealing with critical life events or severe illness (e.g., Badr et al., 2010; Revenson and Lepore, 2012; Rottmann et al., 2015; Falconier and Kuhn) or mental disorders (Bodenmann and Randall, 2013).

More recently, dyadic coping research started to address intercultural aspects in African, American, Asian and European couples (Falconier et al., 2016; Hilpert et al., 2016).

## This Special Issue

The content of the 18 contributions, forming this special issue, ranges from contributions on specific *mechanisms* of dyadic coping (contribution by Pagani et al.; Zietlow et al.), *different types of stressors* (contributions by Canzi et al.; Fallahchai et al.; Molgora et al.), *health or illness issues* (contribution by Acquati and Kayser; Badr et al.; Lameiras et al.; Langer et al.; Martos et al.; Pow et al.; Rentscher; Sallay et al.; Switzer et al.; Tkachenko et al.; Van Schoors et al.), and *novel methods* such as dyadic coping in language use (Lau et al.) up to a *review* article (Falconier and Kuhn), integrating the conceptual and empirical literature on dyadic coping.

## Future Directions

As demonstrated by this special issue, dyadic coping represents an inspiring field of research that spans many topics and disciplines (clinical psychology, health psychology, counseling, family science, developmental and personality psychology). Future studies are encouraged to further integrate multi-methods approaches (self-report, behavior coding, physiology, voice stress, pronoun use etc.) by using longitudinal designs (collection of data over several months or years). Another promising focus is examining overt dyadic coping during everyday interactions of couples at their home, as thus far, little is known about how often and how couples really cope together in everyday life, capturing life as lived (Bolger et al., 2003).

Another focus of dyadic coping research might further address parent-child dyadic coping interactions or adults caring for aging parents, where only few studies were conducted (Zemp et al., 2016).

Furthermore, we do not know much about how dyadic coping evolves over the lifespan of couples (e.g., Johnson et al., 2016), when and it what phase of a couple's life it is particularly relevant and what are long-term effects of dyadic coping on relationship functioning and health.

Finally, more studies on clinical interventions focusing on the improvement of dyadic coping skills, such as the Couples Coping Enhancement Training (CCET) by Bodenmann and Shantinath (2004) or TOGETER by Falconier (2015) are needed. However, current research by Randall and Totenhagen shows promise in this area based on their research with sexual minority individuals experiencing stress due to their marginalized status (Randall et al., 2017a,b; Totenhagen et al., 2018).

## Author Contributions

All authors listed have made a substantial, direct and intellectual contribution to the work, and approved it for publication.

### Conflict of Interest Statement

The authors declare that the research was conducted in the absence of any commercial or financial relationships that could be construed as a potential conflict of interest.
